# The effects of a secondary task on gait in axial spondyloarthritis

**DOI:** 10.1038/s41598-021-98732-z

**Published:** 2021-10-01

**Authors:** Julie Soulard, Jacques Vaillant, Athan Baillet, Philippe Gaudin, Nicolas Vuillerme

**Affiliations:** 1 University Grenoble Alpes, AGEIS, Grenoble, France; 2 CHU Grenoble Alpes, Grenoble, France; 3 University Grenoble Alpes, CNRS, CHU Grenoble Alpes, Grenoble INP, TIMC-IMAG UMR5525, Grenoble, France; 4 Institut Universitaire de France, Paris, France; 5 LabCom Telecom4Health, Orange Labs & Univ. Grenoble Alpes, CNRS, Inria, Grenoble INP-UGA, Grenoble, France

**Keywords:** Rheumatology, Signs and symptoms

## Abstract

Studies on the effects of dual tasking in patients with chronic inflammatory rheumatic diseases are limited. The aim of this study was to assess dual tasking while walking in patients with axial spondyloarthritis (axSpA) in comparison to healthy controls. Thirty patients with axSpA and thirty healthy controls underwent a 10-m walk test at a self-selected comfortable walking speed in single- and dual-task conditions. Foot-worn inertial sensors were used to compute spatiotemporal gait parameters. Analysis of spatiotemporal gait parameters showed that the secondary manual task negatively affected walking performance in terms of significantly decreased mean speed (*p* < 0.001), stride length (*p* < 0.001) and swing time (*p* = 0.008) and increased double support (*p* = 0.002) and stance time (*p* = 0.008). No significant interaction of group and condition was observed. Both groups showed lower gait performance in dual task condition by reducing speed, swing time and stride length, and increasing double support and stance time. Patients with axSpA were not more affected by the dual task than matched healthy controls, suggesting that the secondary manual task did not require greater attention in patients with axSpA. Increasing the complexity of the walking and/or secondary task may increase the sensitivity of the dual-task design to axial spondyloarthritis.

## Introduction

Walking while concurrently performing motor and/or cognitive tasks, such as carrying an object, talking with someone, calling or texting on a smartphone, or other attention-demanding tasks, is the norm rather than the exception during everyday life^1^. Maintaining a safe, stable and efficient gait pattern under such dual task conditions relies on the successful interaction between neural mechanisms that regulate balance and gait control and those that regulate the execution of concurrent motor and/or cognitive task^2^.

There is a plethora of literature reporting that gait pattern is adversely affected during dual-task walking. This is the case for young healthy individuals^2–6^, who generally presented reduced gait speed, shorter stride length, and increased time spent in double limb support in dual-task walking condition compared to single-task walking condition., The negative effect of dual tasks on gait is greater in older adults (e.g., see for reviews^7–10^) and patients populations (patients with neurologic disorders (e.g., see for review^11–14^), suggesting that walking requires greater cognitive resources in these populations *versus* young and/or healthy adults. At this point, it is important to mention that interpreting changes in dual-task walking performance is rather difficult^15^. It is indeed recognised that dual-task interference during walking does depend on a wide variety of factors (e.g., see for reviews^8,10,12,15^), including among others the gait capabilities of the individuals. In fact, what the above mentioned older adults and patient populations seem to have in common is gait disorders. For instance, compared to young adults, older adults walked with slower walking, with shorter steps and prolonged double support (see for review^16^). These age-related changes in spatiotemporal gait parameters are recognised as indicating as the adoption of a more conservative or less destabilising gait, to avoid falls and/or reduce the energetic cost of mobility^16^. Note that this so-called “cautious gait pattern” has also been observed in patients with neurologic disorders (patients after a stroke^17^, patients with sensory or cerebellar ataxia^18^, patients with subcortical disequilibrium^19^).

To the best of our knowledge, no studies have examined dual-task walking in patients with chronic inflammatory rheumatic diseases. However, recent studies have reported similar gait disorders in patients with axSpA^20^ than those observed in the above-mentioned older and patient populations^9,17–19,21,22^. Along these lines, considering also as others^7–14^ that dual task paradigms can provide important insights into the interactions between cognition and the control of walking, the purpose of this study was to assess dual tasking while walking in patients with axSpA in comparison to healthy matched controls.

We hypothesized that (1) the concurrent performance of a secondary manual task would negatively affect spatiotemporal gait parameters during walking (hypothesis 1), and (2) spatiotemporal gait parameters would be more affected by dual-tasking in patients with axSpA as compared to healthy matched controls (hypothesis 2).

## Methods

### Study design

The present study takes part of a larger prospective study called “FOLOMI (Function, Locomotion, Measurement, Inflammation)”^23^, registered in Clinical Trials (NCT03761212), which has been approved by local ethic committee CPP Ile De France 1, RCB: 2017-A03468-45, date of agreement: July 17th, 2018, Last version: V6.0, June 17th, 2020). All research was performed in accordance with the relevant guidelines and regulations. Written signed informed consent was required for all participants of the study to participate in the FOLOMI prospective study^23^.

Data of the present study for the single task condition have been already presented in a previous publication^20^. These data are included only as reference for the dual task walking condition, insofar as the present study focused on the effects of a secondary task on spatiotemporal gait parameters in axSpA. Dual task data have not been published previously in any form.

### Participants

To calculate the number of subjects required for this study, we used results presented in Zebouni et al. study^24^ regarding stride length differences between patients and healthy controls. With a standard deviation of 0.12 and an expected difference of 0.14, and a significance level and a power set respectively at 0.05 and 80%, sample size was estimated at 12 in each group using Sample Size Calculator^25,26^ and was brought to 30 to allow the use of parametric tests.

The first thirty patients with axSpA included in FOLOMI study were age and sex matched to thirty healthy controls. Inclusion and non-inclusion criteria are listed in Table 1^23^.

**Table 1:** Inclusion and non-inclusion criteria for patients with axial spondyloarthritis and healthy controls.

Inclusion criteria	Non-inclusion criteria
**Patients with axSpA**	
Aged 18 to 65 years at time of their first evaluation axSpA (based on ASAS criteria ^51^) or AS (based on modified New York Criteria ^52^) Able to walk 180 m without technical help With stable treatment for 3 months With a public health insurance (French social security)	Musculo-skeletal, cardio-respiratory or neurologic disease that could affect gait Hip or knee arthroplasty done or planned in the following 18 months Not able to speak French Pregnancy or desire of pregnancy in the following 18 months Adults protected by laws (Article L1121-5)
**Healthy controls**	
Aged 18 to 65 years at time of evaluation Able to walk 180 m without technical help With a health insurance	Musculo-skeletal, cardio-respiratory or neurologic disease that could affect gait Hip or knee arthroplasty done Not able to speak French

*axSpA  *axial spondyloarthritis, *AS * ankylosing spondylitis.

### Clinical characteristics of the participants

Clinical characteristics including age, sex, weight, height and pain intensity were gathered for both patients with axSpA and healthy controls by the same observer (JS)^23^.

Disease duration from diagnosis, morning stiffness, the Bath Ankylosing Spondylitis Functional Index (BASFI)^27^, the Bath Ankylosing Spondylitis Disease Activity Index (BASDAI)^27^ and treatments were collected for patients with axSpA only^23^.

### Experimental protocol

A 10-m walk test was performed at comfortable walking speed^28^ in single- and dual-task conditions (3 trials per condition). For the dual-task condition, participants had to walk at comfortable speed and to carry a full cup of water in their dominant hand with the instruction to “perform both tasks as well as possible”^29,30^. Performance of the secondary task was assessed by the examiner who noted whether there was any spillage of water^30^. We decided to use a manual task for the same reasons as those recently explained by Kwon et al. in 2019^5^: “there are more situations that require manual dual task than cognitive dual task in daily living”^5^^(p2)^. Among different manual tasks (carrying a cup, carrying a tray, carrying a tray and a cup), carrying a cup was considered as “more challenging” and related to risk of falling^31^. Thus, carrying a cup is an ecological dual-task, which is commonly performed in daily life^5,31–33^ and is quickly assessed (i.e. there is no requirement to listen/analyse to the records after the execution of the dual task^34,35^) which provides it high usability in clinical practice.

**Figure 1:**
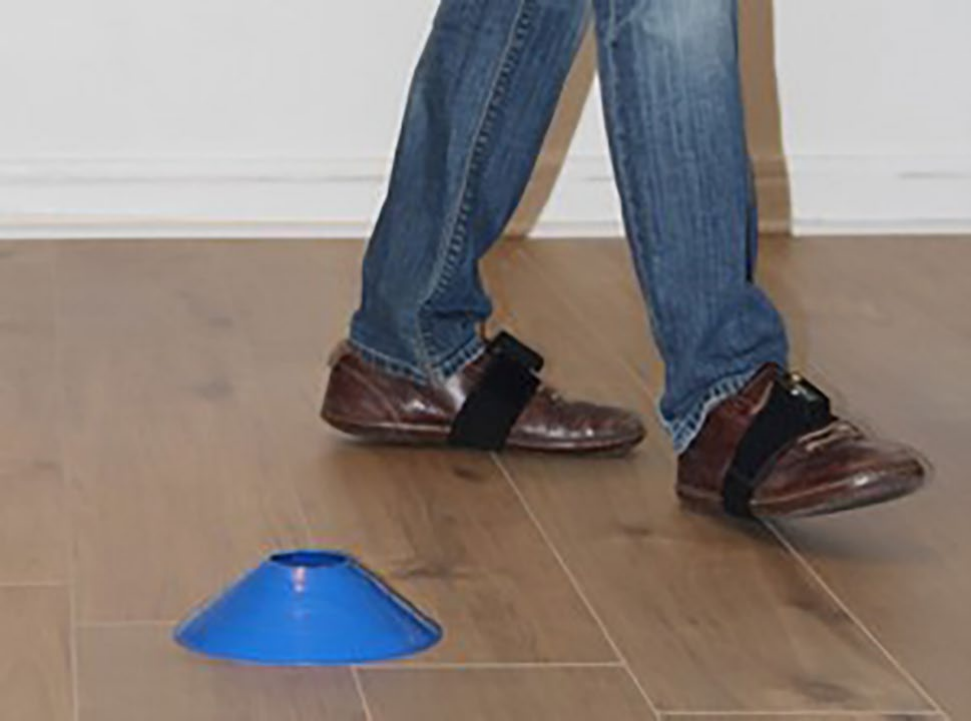
Picture showing the placement of the two foot-worn inertial sensors.

Participants had to wear walking shoes and 2 inertial measurements units (IMUs) with tri-axial accelerometers and gyroscopes (Physilog5, 200 Hz, BioAGM, Gait Up, CH) were placed above both feet (behind the base of the fifth metatarsal)^36^ (Fig. 1). The two first and last steps were removed from the analysis^37,38^ and at least 16 steps were included in the analysis. Gait assessments were performed by the same examiner (JS). For patients with axSpA, assessments were planned at least 2 h from the end of morning stiffness in relationship with possible consequences of morning stiffness on functional limitations^39^.

### Gait outcomes

Spatiotemporal gait parameters were calculated from IMUs (Physilog 5) signals using the Gait Analysis Software (Gait Up, CH, V5.3.0). Means of right and left feet values were computed after checking for non-significant differences between left and right feet. The means of the second and the third trials was calculated for each of the following spatiotemporal gait parameters^40^:

- Speed (m s^−1^): Mean walking stride velocity of forward walking
- Cadence (step/minute): Number of steps in a minute
- Stride length (m): Distance between two consecutive footprints on the ground, from the heel of a foot to the heel of the same foot, one cycle after
- Swing time (%): Portion of the cycle during which the foot is in the air and does not touch the ground
- Stance time (%): Portion of the cycle during which part of the foot touches the ground
- Double support time (%): Portion of the cycle where both feet touch the ground

### Data analysis

Statistical analysis was performed using SPSS 20 (IBM) and Microsoft Excel.

To compare clinical characteristics between patients with axSpA and healthy controls, independent sample t-tests were performed for height, weight and pain intensity. Independent sample t-tests were used to compare performances of the secondary manual task.

To examine the effects of condition and group on spatiotemporal gait parameters, repeated measures analyses of variance (RM-ANOVA) were conducted with within factor being condition (single- or dual-task condition) and between factor being the group (healthy controls or patients with axSpA). From each comparison, 95% confidence intervals (CI) were calculated and effect sizes were computed using partial Eta-squared (η^2^) which was calculated as the ratio of the effect variance to the total variance. A partial η^2^ value of 0.45 means that the independent variable has 45% effect on the dependent variable outcome. The significance of the *p *value was set at 0.05.

## Results

### Study population

The population included in the present study is the same that has been presented in a previous publication and is presented in Table 2^20^.

**Table 2:** Patients with axSpA and sex and age-matched healthy controls clinical characteristics.

Clinical characteristics	Healthy controls (n = 30)	Patients with axSpA (n = 30)	Independent t-test		
			t	*p *value	95% CI (LB-UB)
Age (years), mean ± sd	45.70 ± 10.60	45.37 ± 10.54			
Gender (Male), n (%)	20 (66.6)	20 (66.6)			
Weight (kg), mean (sd)	70.25 ± 10.27	74.15 ± 12.94	− 1.294	0.201	(− 9.94 to 2.13)
Height (cm), mean (sd)	174.47 ± 7.48	170.77 ± 7.82	1.873	0.066	(− 0.25 to 7.65)
Self-reported pain intensity scores at time of evaluation, mean (sd)	0.20 ± 0.66	3.12 ± 2.38	− 6.463	**< 0.001**	(− 3.82 to − 2.02)
HLA-B27 status, n (%)		Positive: 18 (60.0) Negative: 9 (30.0) Unknown: 3 (10.0)			

*axSpA *axial spondyloarthritis, *sd *standard deviation, *n *number, *LB *lower bound, *UB *upper bound.

Mean disease axSpA duration was of 11.77 ± 10.11 years and mean morning stiffness duration of patients with axSpA was 28.17 ± 33.71 min. Patients with axSpA presented low disease activity (mean BASDAI: 3.04 ± 1.90) and low impact of axSpA on physical function (mean BASFI: 2.86 ± 2.04). Most of patients with axSpA had anti-TNF treatment (n = 21, 70.0%), while others had Interleukin-17A (n = 2, 6.7%), nonsteroidal anti-inflammatory drugs (n = 7, 23.3%), disease modifying anti-rheumatic drugs (n = 3, 10.0%) and/or pain reliefs (n = 7, 23.3%).

### Gait performance

**Figure 2:**
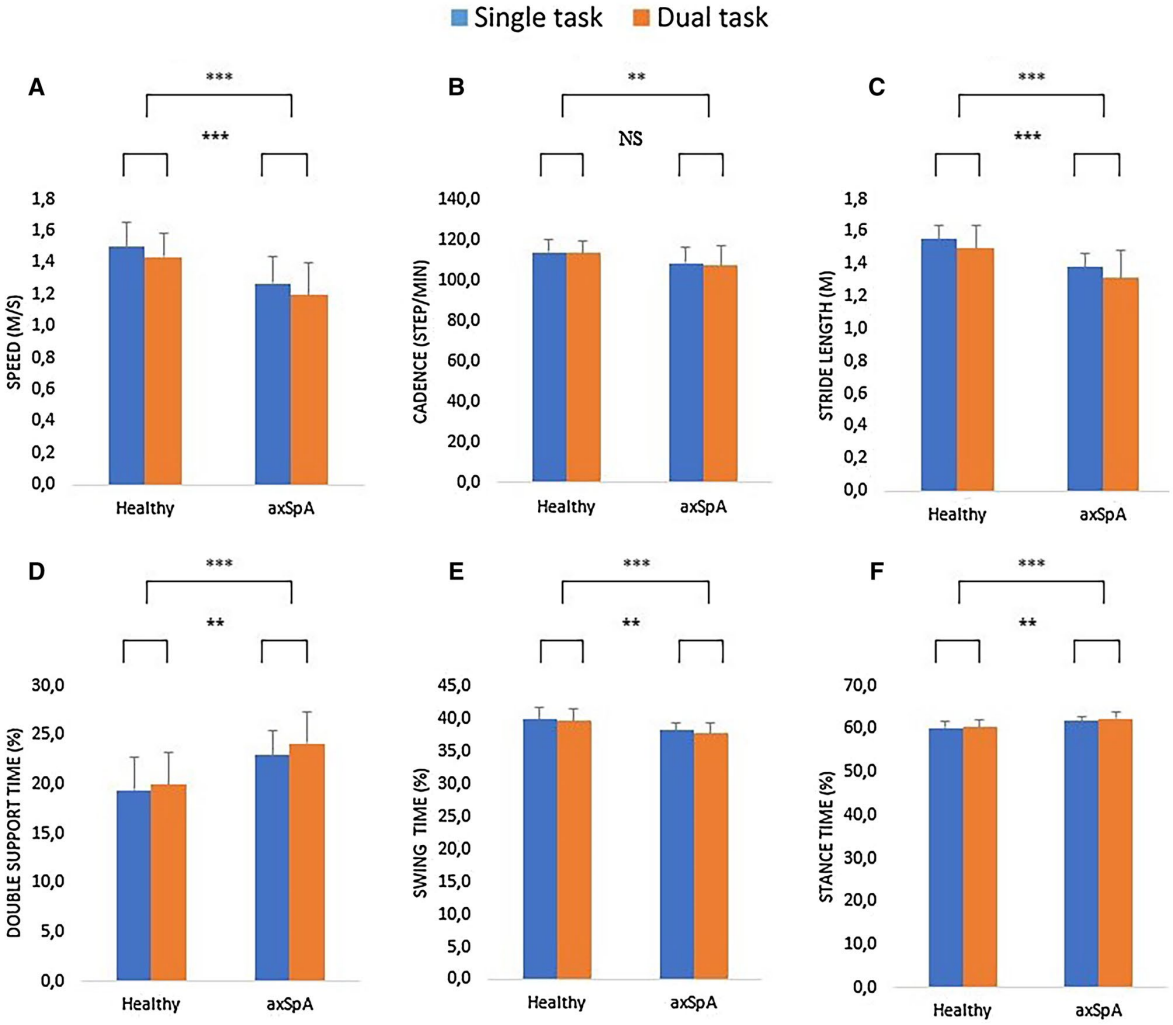
Mean and standard deviation of the spatiotemporal gait parameters obtained in healthy controls and patients with axSpA in single- and dual-task conditions (**A** gait speed, **B** cadence, **C** stride length, **D** double support time, **E** swing time, **F** stance time) (**p < 0.01, ***p < 0.001, *NS* non significant).

Spatiotemporal gait parameters in single- and dual-task conditions are illustrated in Fig. 2 for each group.

Results showed main effects of group for each evaluated spatiotemporal gait parameters, namely gait speed (F(1,58) = 31.528, *p* < 0.001, partial η^2^ = 0.352, Fig. 2A), cadence (F(1,58) = 9.383, *p* = 0.003, partial η^2^ = 0.139, Fig. 2B), stride length (F(1,58) = 23.067, *p* < 0.001, partial η^2^ = 0.285, Fig. 2C), double support time (F(1,57) = 24.329, *p* < 0.001, partial η^2^ = 0.299, Fig. 2D), swing time (F(1,58) = 20.922, *p* < 0.001, partial η^2^ = 0.265, Fig. 2E), and stance time (F(1,58) = 20.922, *p* < 0.001, partial η^2^ = 0.265, Fig. 2F).

Results also showed main effects of condition for five out of six evaluated spatiotemporal gait parameters, namely, gait speed (F(1,58) = 23.674, *p* < 0.001, partial η^2^ = 0.290, Fig. 2A), stride length (F(1,58) = 42.833, *p* < 0.001, partial η^2^ = 0.425, Fig. 2C), double support time (F(1,57) = 11.105, *p* = 0.002, partial η^2^ = 0.163, Fig. 2D), swing time (F(1,58) = 7.591, *p* = 0.008, partial η^2^ = 0.116, Fig. 2E), and stance time (F(1,58) = 7.691, *p* = 0.008, partial η^2^ = 0.116, Fig. 2F). No significant main effect of condition was observed for cadence (F(1,58) = 1.865, *p* = 0.177, partial η^2^ = 0.031, Fig. 2B).

Results further showed no significant interaction of group and condition for all evaluated spatiotemporal parameters, namely gait speed (F(1,58) = 0.108, *p* = 0.743, partial η^2^ = 0.002), cadence (F(1,58) = 0.540, *p* = 0.465, partial η^2^ = 0.009), stride length (F(1,58) = 0.215, *p* = 0.644, partial η^2^ = 0.004), double support time (F(1,57) = 1.562, *p* = 0.216, partial η^2^ = 0.027), swing time (F(1,58) = 1.171, *p* = 0.284, partial η^2^ = 0.020), and stance time (F(1,58) = 1.171, *p* = 0.284, partial η^2^ = 0.020).

### Secondary task performance

Analysis of the performance of the secondary task first showed that most of the participants did not spilled water out of the glass (axSpA: n = 29, 96.6%; controls: n = 28, 93.3%). Furthermore, no significant difference was found on the number of time the participants spilled water out of the glass between the two groups (axSpA: 0.13 ± 0.73, controls: 0.07 ± 0.25, *p* = 0.638).

## Discussion

Dual-task paradigm has become a well-established research paradigm to examine the interactions between cognition and the control of walking (e.g. see ^2–14^, for recent reviews). Although this paradigm has been widely used in older adults^7–10^ and patient populations^11–14^, to the best of our knowledge, this is the first study assessing dual tasking while walking in patients with axSpA^41^.

Results first showed that gait performance was adversely affected by the concurrent execution of the manual task in healthy adults. Specifically, significant changes in spatiotemporal gait parameters were characterized by walking speed decreased by 4.8%, stride length decreased by 3.9%, swing time decreased by 0.6%, stance time increased by 0.4% and double support time increased by 2.7% during the dual-task as compared to the single-task walking condition. These results were expected in line with hypothesis 1. They are indeed in accordance with previous studies that have used a similar secondary manual task while walking at self-selected comfortable speed^5,42,43^ and have reported significant alterations of gait patterns in healthy individuals^5,42,43^. These results ^5,42,43^ and ours hence suggest that interference of manual task with gait performance, that is classically explained by competing demands for attentional resources involved in both concurrent tasks^3,8,10^, can occur even in healthy adults^5,42,43^. Otherwise, indeed, the simultaneous execution of the manual task would not have significantly affected gait performance or secondary manual task performance.

More originally, in line with our hypothesis 1, results further showed that walking performance of patients with axSpA also was significantly impaired while performing walking and manual task simultaneously. The concurrent execution of the secondary manual task negatively affected walking performance in terms of significantly decreased mean speed by 6.5%, stride length by 5.2%, and swing time 1.4%, and significantly increased double support by 4.8% and stance time by 0.9% in the dual-task condition as compared to the single-task condition. To the best of our knowledge this result obtained in patients with axSpA is new.

Finally, perhaps the most interesting (and unexpected) result of this study was that dual task effects on walking performance were similar between patients with axSpA and age and sex matched healthy controls. Indeed, no significant interaction of group and condition for any of the calculated spatiotemporal gait parameter were found. In other words, contrary to our hypothesis 2 and to previous observations in other patient populations (e.g. patients with neurologic disorders (e.g., see for reviews^11–14^)), patients with axSpA were not more affected by the dual task than age and sex matched healthy controls. Interestingly, together with the absence of significant difference on the performance of the secondary manual task between the two groups, this result suggests that gait, although significantly altered in patients with axSpA in both single- and dual-task conditions as compared to age and sex healthy controls, did not require greater attention in patients with axSpA.

That being said, it is important to bear in mind that the explanations of dual-task interference are based on the assumption that attentional resources are limited^3^. Accordingly, dual-task interference is likely to occur if the available central capacity of the individual is exceeded, which causes an inability to appropriately adapt the allocation of attention between the two concurrently performed gait and secondary tasks. Accordingly, dual-task interference can theoretically be either (1) cognitive-locomotor related (i.e. with effects on *both* cognitive and locomotor tasks), (2) cognitive-related (i.e. with effects on cognitive task *only*), (3) motor-related (i.e. with effects on locomotor task *only*) or (4) absent^12^. In the present study, dual-task interference observed in both patients with axSpA and healthy controls can be placed in the class of ‘motor-related’ as the gait performance was significantly altered during dual-task walking and the performance of secondary manual task while walking remained maximal. However, note that both patients with axSpA and healthy controls experienced little ‘motor-related’ dual-task interference, as the decrement in gait performance on the a 10-m walk test relative to single-task performance was minimal (see percentage changes in spatiotemporal gait parameters from single- to dual-task condition below, from 0.4 to 4.78% in healthy controls and from 0.87 to 6.5% in patients with axSpA).

At this point, within the context of a dual-task, the attentional demand associated with gait depends on various factors^8,10,12,15^ that could account for the observed results. These factors could be regarded as possible explanations and as study limitation that we acknowledge.

Firstly, we are aware that the type, the level of complexity and novelty of either the primary walking task^33,44–46^ and/or concurrent secondary task^8,10,12,15^ can significantly influence the dual-task interference during walking (e.g., see ^8,10,12,15^ for recent reviews). However, in the present study, only one walking task (walking at a self-selected comfortable speed)^28,45^ and one secondary task (‘carrying a full cup of water’)^5,42^ were investigated. These two tasks were chosen on the basis on the recommendations from a recent systematic review that dual-task assessments should be performed “in similar contexts of individuals' daily lives to ensure ecological validity” (^12^, page 1). Note however that these two tasks may not be representative of all dual-task conditions during walking. These two tasks hence represents relatively easy and familiar tasks in terms of level of complexity (i.e. the task’s constraints and environmental context) and novelty (i.e. the individual’s previous experience with performance of the tasks). What is more, these two tasks are also routinely used in clinical settings to assess gait in a wide range of patient populations. Increasing tasks complexity could thus have modify tasks performance with possible differences between patients with axSpA and healthy controls during dual-tasking.

Secondly, we are aware that dual-task interference during walking also depends on the study population^47,48^. Naturally, this factor must be considered in close connection with the above-mentioned task-related factors. Indeed, depending on the characteristics of the study population, the tasks may not be challenging enough to reach/exceed the central capacity limit. It is thus probable that the attentional resources required to simultaneously perform the walking task and the manual task did not overload the available central resources and, consequently, only induced little dual-task interference with minor gait alterations. To the best of our knowledge, the present study is the first to investigate the effects of dual tasking on gait in patients with chronic rheumatic disease. One study assessed previously the effects of an arithmetic task on postural control in patients with rheumatoid arthritis^49^ and found that the effect of the arithmetic task on balance parameters was small and similar in patients and controls. These authors hypothesized that the slow evolution of joint destruction may let time to patients to adapt their postural coordination pattern without requiring attentional control^49^. A similar interpretation could be argue in the present population. However, the authors visually found that patients with rheumatoid arthritis with most severe joint destruction showed relatively strong dual-task effect and may have not have enough sample size to see this effect statistically^49^. Patients of the present study may not represent the whole population of axSpA^50^, as theywere 18–65, with stable treatment for at least 3 months, able to walk 180 m without technical help. Besides, they had low disease activity (BASDAI: 3.04 ± 1.90) with low impact of physical function (BASFI: 2.86 ± 2.04).

In other words, the present findings are neither transferable to other walking and secondary tasks nor other cohorts of patients with axSpA. Accordingly, further studies that will employ other walking tasks^33,44–46^ and/or secondary tasks^8,10,12,15^ and/or recruit other cohorts (e.g., patients with lower walking range, or higher disease activity with more impact on physical function) are needed to confirm and to generalize our results. It is possible that increasing the complexity of the walking and/or secondary task would increase the sensitivity of the dual-task design to patients with chronic rheumatic diseases. Limitations notwithstanding, the findings of the present study showed for the first time that gait of patients with axSpA is significantly impaired while performing walking and manual tasks simultaneously similar to what was observed in healthy controls.

### Ethics approval and consent to participate

The study was approved by local ethic committee (CPP IDF1. RCB: 2017-A03468-45. Date of agreement: July 17th. Last version: V6.0. 2020. June 17th). The study is registered on ClinicalTrials.gov. With the following ID: NCT03761212 and followed the SPIRIT checklist. Written informed consent were obtained from all participants by the physiotherapist or a doctor.

### Patient involvement

Patients were recruited from rheumatologist. Results will be disseminated via email to all study participants and via conference presentations to rheumatologists, general doctors, physical therapists and researchers. A poster of the results will be displayed in the Rheumatology Department of Grenoble University Hospital (France) to inform all patients with axSpA and visitors on the results of the study.

## Data Availability

The data of the present manuscript can be available on demand to the corresponding author.
